# Influence of the dilution method on the intraocular duration of C_3_F_8_ in vitrectomy for macular hole: a randomized clinical trial

**DOI:** 10.5935/0004-2749.2022-0336

**Published:** 2024-02-23

**Authors:** Alexandre Paashaus da Costa Pinto, Daniel Tenório Camelo Soares, Marília Rocha Costa, Rodrigo Pessoa Cavalcanti Lira

**Affiliations:** 1 Universidade Federal de Pernambuco, Recife, PE, Brazil

**Keywords:** Retinal perforations/surgery, Vitrectomy, Sulfur hexafluoride/administration & dosage, Fluorocarbons/administration & dosage, Gases, Tomography, optical coherence

## Abstract

**Purpose:**

To compare the injection of small amounts of undiluted C_3_F_8_ with the traditional gas injection in vitrectomy for macular hole treatment.

**Methods:**

This clinical trial included 26 individuals divided into two groups. Group 1 received an intravitreal injection of 0.9-1.0 mL of 100% C_3_F_8_, and Group 2 received 15-20 mL of 20% C_3_F_8_.

**Results:**

The median intraocular gas duration was 31 days in Group 1 and 34 in Group 2. The median letter gains in corrected distance visual acuity for the 26^th^ postoperative week were 20 letters in Group 1 and 12.5 in Group 2. The median intraocular pressure was normal in both groups. Primary anatomical success was 11/13 in both groups.

**Conclusions:**

The use of C_3_F_8_ gas in a small undiluted volume is an alternative that slightly reduces the duration of the gas without negatively affecting the anatomical and visual response.

## INTRODUCTION

A macular hole (MH) is a full-thickness defect of the foveal retina, causing blurred vision, metamorphopsia, and central scotoma. Most cases are idiopathic. The most accepted pathophysiology is that there is an abnormal vitreomacular traction (VMT) that results in a centrifugal movement of photoreceptors^(1)^, inflammatory cell proliferation, and fibrosis^(2)^.

If the individual has a symptomatic MH, surgery is recommended. Pars plana vitrectomy (PPV), the removal of cortical vitreous and epiretinal membranes, and face-down intraocular gas tamponade, is the treatment of choice^(3)^. The most used gases are perfluoropropane (C_3_F_8_) and sulfur hexafluoride (SF_6_) which have similar efficacy^(4)^. SF_6_ produces similar clinical outcomes to C_3_F_8_ for primary closure and visual outcomes; however, C_3_F_8_ is preferred for difficult MH cases, such as large MH and MH retinal detachment in myopic patients^(5)^.

C_3_F_8_ is used at a concentration between 12% and 18% by intravitreal injection of large volumes of pre-diluted gas^(4)^. This study evaluated the duration of intraocular gas in vitrectomy for MH by comparing the injection of small amounts of undiluted C_3_F_8_, which would be more economical, with the traditional injection of larger volumes of pre-diluted C_3_F_8_.

## METHODS

This partially blinded randomized controlled clinical trial included 26 individuals who underwent PPV for MH between 2019 and 2020, in Recife, Brazil. It was approved by the Research Ethics Committee of *Hospital das Clinicas at Universidade Federal de Pernambuco* (CAAE no. 12347319.9.0000.8807). All patients signed an informed consent form. The study was registered under the name “Influence of Preparation Method on Gas Duration in Vitrectomy for MH;” Clinicaltrials.gov Identifier No: NCT04527848; http://clinicaltrials.gov/ct2/show/NCT04527848.

The inclusion criteria were: pseudophakia, 50 years of age or above, no previous vitreoretinal surgery, a diagnosis of full-thickness MH by optical coherence tomography (OCT). The exclusion criteria were: allergy to any product used during the procedure, planned airplane travel in the first 60 postoperative days, myopia >6 diopters or axial length >26 mm, history of eye trauma, retinal dystrophy, retinal detachment, abnormal eye shape, glaucoma, diabetic retinopathy, or other eye disease.

**Figure 1:**
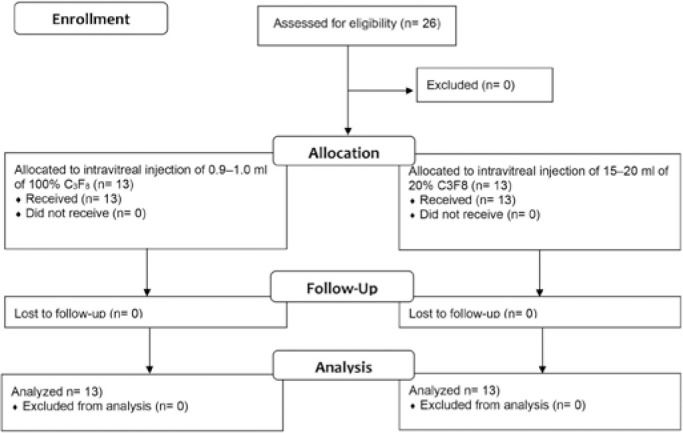
Profile of the randomized clinical trial.

The participants were randomly assigned to one of two groups. Group 1 received an intravitreal injection of 0.9-1.0 mL of 100% C_3_F_8_ (Alcon Laboratories^®^, USA), and Group 2 received an intravitreal injection of 15-20 mL of 20% C_3_F_8_, giving a final intravitreal concentration of 12%-18% in all individuals (Figure 1).

Before surgery, the axial length (AL) was measured using optical biometry (IOLMaster^®^ 500, Carl Zeiss^®^, Germany). All surgeries were performed by the same surgeon. A full PPV with vitreous base shaving was performed using the Constellation^®^ vitrectomy system (Alcon Laboratories, USA) with 23-gage vitrectomy probes, 3 ports, and valve trocars inserted into the sclera 3.5 mm from the limbus. Complete fluid-air exchange was performed. Afterwards, the air infusion pressure was adjusted to 10 mmHg to maintain the ocular volume (the air was injected through the inferior temporal valve trocar). The vitreous chamber was filled with Brilliant Blue G (Opht-Blue^®^, Ophthalmos Rohto^®^, Brazil) through the temporal superior valve trocar. It was diluted to a concentration of 0.005% using a 10-mL syringe with a 0.2-mL increment. Next, the Brilliant Blue G was replaced with balanced salt solution. Then, the internal limiting membrane peeling, and full fluid-air exchange was conducted.

At the end of the surgery, the C_3_F_8_ was aspirated through a sterile disposable three-way tap coupled to sterile filters, and to a 1 mL syringe with increments of 0.02 mL in Group 1 or to a 20 mL syringe with increments of 1 mL in Group 2. As physiological dead space exists within the system, the air contained within these spaces may affect accuracy. Pure gas was drawn from the cylinder to ensure complete evacuation of air from the dead space. The appropriate amount of pure gas was then drawn into the syringe (0.9-1.0 mL in Group 1, and 3-4 mL in Group 2). In Group 1, after removing one of the three trocars, the syringe with one filter was disconnected and the entire content was injected through one of the valve trocars, with passive extrusion of the excess volume through a cannula in the second valve trocar (direct air-gas dilution). The two residual trocars were later removed. In Group 2, after removing one of the three trocars, the syringe with one filter was disconnected, and the three-way tap was turned to the other unused filter. Then, 12-16-mL air was drawn in to achieve the appropriate air-gas mixture, which was injected through one of the valve trocars, with passive extrusion of the excess volume through a cannula in the second valve trocar. The two residual trocars were later removed. Perfusion of the central retinal artery was checked at the end of the procedure by indirect ophthalmoscopy. Paracentesis was performed if there was evidence of ocular hypertension. Postoperatively, the patient was instructed to maintain a face-down posture for 7 days.

The AL was used to estimate the volume of the vitreous chamber^(6)^. The amount of C_3_F_8_ injected intravitreally was calculated based on the estimated volume of the vitreous cavity. In a pseudophakic individual with a 24 mm AL, the volume of the vitreous cavity is approximately 5.2 mL. In Group 1, the volume of 100% C_3_F_8_ injected was 0.9 mL and 1.0 mL for AL <24 mm and ≥24 mm, respectively. In Group 2, the volume of 20% C_3_F_8_ was 15 mL (3 mL 100% C_3_F_8_ + 12 mL air) and 20 mL (4 mL 100% C_3_F_8_ + 16 mL air), respectively. The intravitreal concentration of C_3_F_8_ was calculated using the dilution formula (initial concentration × initial volume = final concentration × final volume).

Data were collected using a medical history form completed by the physician during the first medical examination. Corrected distance visual acuity (CDVA) based on the Early Treatment Diabetic Retinopathy Study (ETDRS) charts, and biomicroscopy, tonometry, indirect ophthalmoscopy, OCT, and medical events were recorded on a standardized form by a researcher blinded to the treatment.

The classification of full-thickness MH was based on the International Vitreomacular Traction Study Group criteria^(7)^. BM size was based on the horizontal diameter at the narrowest point using the OCT compass function.

The primary endpoint was the intraocular duration of C_3_F_8_ (days). Secondary outcomes were the gain of letters in the CDVA on the 26th week, intraocular pressure (IOP) on the first postoperative day and 26^th^ week, and the anatomical success (complete closure of the macula hole in the OCT after the C_3_F_8_ bubble had disappeared). The patient was instructed to notify the researchers immediately when they no longer noticed the presence of the gas (the gas bubble, easily perceived by the individual, was noticed as an inferior, shrinking, mobile scotoma). An additional evaluation was carried out, preferably on the same day, to confirm the absence of gas.

The 0.9-1.0 mL dose of 100% C_3_F_8_ cost approximately US$30 and the 15-20 mL dose of 20% C_3_F_8_ cost US$120.

Subjects were randomized in a 1:1 ratio. The groups were stratified by gender, and block sizes of 4 were used. Two individuals from each block were allocated to each group. One nurse generated the random allocation sequence, and another nurse enrolled and assigned the participants to the interventions in a blinded fashion. The surgeon was not masked because the gas injection syringes were of different sizes for each group. The physician who conducted the postoperative evaluations was masked. No participant was lost to follow-up or withdrew from the study.

A sample size of 13 individuals per group was calcu-lated assuming that the study would have a power greater than 80% and a probability of type 1 error less than 0.05% (two-tailed) to detect a difference of 4 days in the intraocular duration of the gas (with a standard deviation of 3 days, and allocation radius of 1:1) between the groups.

Data were summarized using descriptive statistics. The Kolmogorov-Smirnov normality test was used to evaluate the distribution of continuous data. Means and standard deviations (SD) were used for normally distributed data and medians and interquartile ranges (IQRs) for non-normally distributed data. Between-group diffe-rences in continuous variables were compared using the independent samples *t-test* for normally distributed data or the Mann-Whitney *U*-test for non-normally distributed data. Categorical variables were compared using the χ^2^ test or Fisher’s exact test when appropriate. The intravitreal duration of the gas was assessed with the Kaplan-Meier survival analysis log-rank test. The analyses were performed using SPSS version 21 (IBM Corporation, Armonk, NY, USA). The p-values were two-tailed, and statistical significance was set at 0.05.

## RESULTS

The sample consisted of 26 patients who underwent PPV for MH (Table 1). The mean (SD, range) age was 71 years (5, 58-79). Fifteen individuals (57.7%) were women. Surgery was performed on the right eye in 13 (50%) individuals. The mean (SD, variation) MH size was 332 mm (112, 132-488). The MH was small in 9 (34.6%) individuals, medium in 9 (34.6%) individuals, and large in 8 (30.8%) individuals. The mean (SD, variation) AL was 24.02 mm (0.70, 22.92-25.26), vitreous cavity volume was 5.22 mL (0.50, 4.43-6.09), and C_3_F_8_ intravitreal concentration was 15.23% (0.49, 14.10-16.06). The median (IQR, variation) preoperative CDVA was 43 (11, 38-53) letters (± 20/160; Snellen) and preoperative IOP was 15 mmHg (7, 11-18).

**Table 1:** Preoperative data according to the intervention group

	Group 1 0.9-1.0 mL 100% C3F8	Group 2 15-20 mL 20% C3F8	p-value
Age (years) ^a^	72 ± 4	70 ± 6	0.397^c^
Female	7/13	8/13	0.691^d^
Right eye	7/13	6/13	0.695^d^
Macular hole size (µm) ^a^	333 ± 107	330 ± 123	0.952^c^
Macular hole classification:			0.895^d^
Small (<250 µm)	4/13	5/13	
Medium (250-400 µm)	5/13	4/13	
Large (>400 µm)	4/13	4/13	
Axial length (mm)^a^	24.01 ± 0.81	24.04 ± 0.61	0.918^c^
Vitreous cavity volume (mL)^a^	5.21 ± 0.58	5.23 ± 0.43	0.918^c^
Concentration of intravitreal gas (%)^a^	15.1 ± 0.6	15.3 ± 0.3	0.341^c^
Preoperative CDVA (letters)^b^	43 (15)	43 (10)	0.342^e^
Preoperative CDVA (Snellen)	± 20/160	± 20/160	
Preoperative IOP (mmHg)^b^	15 (1)	14 (2)	0.254^e^

a = mean ± standard deviation;

b = median (interquartile range);

c = independent samples t-test;

d = chi-square test;

e = Mann-Whitney *U*-test. CDVA= corrected distance visual acuity (based on ETDRS charts); IOP = intraocular pressure.

**Figure 2:**
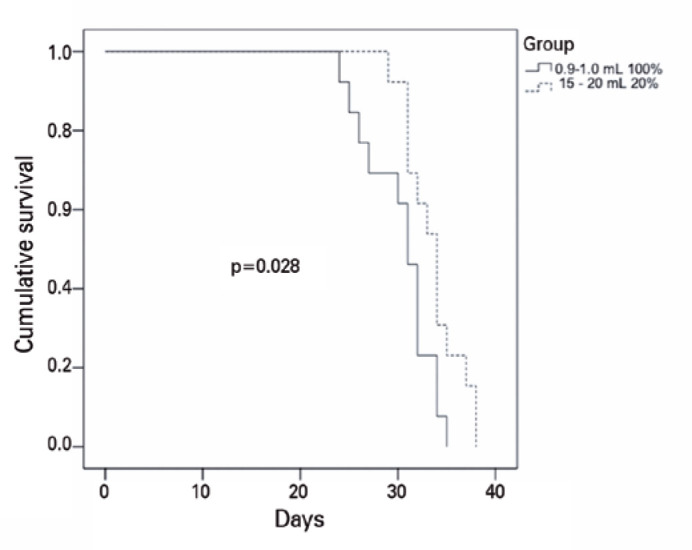
Kaplan-Meier curve of C_3_F_8_ intraocular duration (days) after vitrectomy for macula hole (injection of 0.9-1.0 mL of 100% C_3_F_8_ versus 15-20 mL of 20% C_3_F_8_).

The median (IQR; variation) intraocular gas duration (Figure 2) was 31 days (7; 24-35) in Group 1 and 34 days (5; 29-38) in Group 2 (p=0.028; log-rank test). The median (IQR; variation) letter gains in CDVA for the 26^th^ postoperative week were 20 letters (25; 0-25) in Group 1 and 12.5 letters (35; 0-35) in Group 2 (p=0.801). The median (IQR; variation) IOP on the 1^st^ postoperative day was 13 mmHg (3; 6-24) in Group 1 and 12 mmHg (5; 9-22) in Group 2 (p=0.448). The median (IQR; variation) IOP at the 26^th^ postoperative week was 16 mmHg (3; 12-19) in Group 1 and 14 mmHg (6; 12-18) in Group 2 (p=0.418).

The primary anatomical success was 11/13 (84.6%) in Group 1 and 11/13 (84.6%) in Group 2 (p=0.999). Regarding adverse medical events: 2/13 (15.4%) individuals in Group 1 and 1/13 (7.7%) individuals in Group 2 presented ocular hypertension (IOP above 21 mmHg) on the first postoperative day (p=0.999).

## DISCUSSION

As far as we know, this study is the first to compare diluted versus undiluted C_3_F_8_ for MH surgery. Except for the shorter intraocular duration when injected in a small undiluted volume, other properties of C_3_F_8_ related to visual acuity gain, MH closure in OCT, and adverse effects were unchanged.

The intraocular gases used in surgery act to buffer and seal the macula. The gas also forms a mold for migrating glial cells and promotes the healing of the hole^(8)^. It was believed that a longer intraocular persistence of gas would give better surgical results. However, recent studies have shown that the MH closure occurs between the first and seventh postoperative day^(9-13)^. Additionally, the gas bubble limits the individual’s vision while it persists, adversely affecting daily activities. This clinical trial showed that the use of concentrated doses and less C_3_F_8_ slightly reduced the gas duration without negatively affecting the anatomical and visual response. Of note, regarding healing, Essex et al.^(13)^ compared the outcomes between patients who were not advised to adopt a postoperative face-down position and those who were, and found no difference for holes of less than 400 µm in diameter.

Most studies show that MH closure rates range from 85% to 100%^(8,13-16)^. This study showed a similar rate of MH closure in both groups.

The best-corrected visual acuity (BCVA) improvement was statistically similar in both groups. Visual acuity improvement following MH surgery usually ranges from 2.7 to 6.9 ETDRS lines^(4,5)^.

Neither gas presentation was associated with an increased risk of adverse events. Most studies reported similar results for shortand long-term complication rates^(15,17)^.

The following limitations must be considered. First, the study used 23-gage vitrectomy; however, there is a tendency to migrate to smaller sclerotomies. It may be important to conduct a study using 25 or 27-gage, which would provide greater accuracy as there is, theoretically, less gas leakage through incisions immediately after surgery. However, based on the results of Dihowm, MacCumber^(18)^, 20, 23, and 25-gage PPV have similar MH closure rates and visual acuity outcomes. Second, the sample size was insufficient to perform subgroup analysis, for example, according to the size of the MH. The third limitation is that this study did not evaluate eyes with degenerative myopia or nanophthalmos. The fourth limitation is that the results are valid only for C_3_F_8_. Finally, this study did not measure cataract formation as all patients were pseudophakic.

The intraocular duration of C_3_F_8_ was shorter when injected in a small undiluted volume without affecting the clinical or anatomical success of the treatment. Therefore, in eyes between 21 and 26 mm of LA, the use of gas in a small undiluted volume may be an option since its cost was three times less than the diluted form.
